# The Effect of Sodium Humate on Sheep In Vitro Fermentation Characteristics and Rumen Bacterial Community

**DOI:** 10.3390/microorganisms13061266

**Published:** 2025-05-29

**Authors:** Na Yin, Yuchao Hu, Xiangting Cai, Long Gao, Wenwen Wang, Yuan Wang, Jingwei Qi

**Affiliations:** 1 College of Animal Science, Inner Mongolia Agricultural University, Hohhot 010018, China; yinna0420@163.com (N.Y.); yuchaohu1994@163.com (Y.H.); 15149846460@163.com (X.C.); gaolong0623@163.com (L.G.); wangwenwen2017@emails.imau.edu.cn (W.W.); wangyuan.926@163.com (Y.W.); 2 Inner Mongolia Herbivorous Livestock Feed Engineering Technology Research Center, Hohhot 010018, China

**Keywords:** sodium humate, fermentation parameters, in vitro, rumen bacteria, sheep

## Abstract

This study aimed to determine the optimal supplementation level of sodium humate (SH) for improving rumen fermentation efficiency in vitro. Using rumen fluid from four donor ewes with three experimental replicates per treatment, we evaluated a basal diet supplemented with SH at 0 (control), 0.5 (SH0.5), 1 (SH1), and 2 (SH2) g/kg dry matter. The results of this study revealed that after 12 h of incubation, compared to the control group, the SH0.5 group significantly decreased gas production (GP) by −11.66% (*p* < 0.01). There were no significant differences in pH values, bacterial crude protein (BCP) content, and ammonia nitrogen (NH₃-N) concentration among the groups (*p* > 0.05). After 24 h of incubation, no significant differences in pH values were observed among the groups (*p* > 0.05). The SH1 group exhibited significantly higher BCP content compared to other treatments (*p* < 0.05), concomitant with a marked reduction in NH₃-N concentration (*p* < 0.01). Compared to the control group, GP in the SH1 group increased significantly by 7.16%, and a significant increase of 5.43% (*p* < 0.05), while it decreased significantly by −9.96% in the SH0.5 group (*p* < 0.01). However, no significant differences in volatile fatty acids were observed among the groups after either 12 or 24 h of fermentation. The addition of 1 g/kg SH altered the composition of the rumen bacterial community, which was indicated by the increased relative abundances of *Prevotella, Anaerovibrio,* and *Saccharofermentans* and the decreased relative abundances of Actinobacteriota, *Lachnospiraceae_NK3A20_group*, and *[Ruminococcus]_gauvreauii_group* (*p* < 0.05). Furthermore, *Anaerovibrio* was negatively correlated with NH₃-N and positively correlated with gas production, while *[Ruminococcus]_gauvreauii_group* was negatively correlated with gas production. The study indicates that the addition of 1 g/kg SH optimizes rumen fermentation efficiency and improves nutrient utilization by modulating the structure and composition of the bacterial community, thus serving as an effective additive for enhancing rumen fermentation and feed utilization in ruminants.

## 1. Introduction

In recent years, in pursuit of enhanced production efficiency, sheep were often provided with high-concentrate diets. However, long-term feeding of such diets may adversely affect rumen function, including disruptions to bacterial balance and fermentation patterns [1]. To address these challenges, supplementation of ruminant diets with additives has emerged as a promising strategy to modulate rumen fermentation and microbiota structure, thereby improving rumen health [2,3].

Among these additives, humic substances (HSs), including humic acids (HAs) and fulvic acids (FAs), are organic macromolecules derived from decomposed plant and natural residues, widely distributed in soils, aquatic systems, and sediments [4]. Due to their anti-inflammatory, antibacterial, and antioxidant properties, HSs have been utilized in traditional medicine for millennia [5,6]. Notably, HSs form a protective film on the mucous epithelial cells of the gastrointestinal tract against infections and toxins; their large colloidal structure ensures good shielding of the gastrointestinal mucosa, peripheral capillaries, and damaged mucous cells. As a result of this process, the re-absorption of toxic metabolites is reduced or completely prevented, especially after infections or in the presence of harmful substances in animal feed [7]. For instance, Murbach et al. (2020) [8] demonstrated that dietary supplementation with 0.5 g/kg HAs significantly improved the health status of monogastric animals by mitigating inflammation, a mechanism particularly beneficial in the presence of feed-borne toxins or post-infection recovery. Beyond their protective role, HSs have been shown to improve feed efficiency and growth performance. In poultry, the addition of HSs at a dose of 0.2 g/kg in the diet increased egg production and quality [6]. In pigs, HSs at a dose of 0.3 g/kg improved growth rates and feed conversion efficiency [9].

Sodium humate (SH), a salt of HAs, has been used as a therapeutic agent for dyspepsia, diarrhea, and acute intoxication in animals, with potential antimicrobial, antiviral, anti-inflammatory, antidiarrheal, and antirheumatic properties [10]. As a result, it may enhance immunity, promote growth, improve the feed conversion rate, and reduce nitrogen losses, making it a useful feed additive in livestock production [11,12]. Research shows that adding 1 g/kg SH can reduce diarrhea incidence [13]. SH has also been shown to enhance feed conversion efficiency by 15% in dairy cows at a dose of 1.5 g/kg [14]. However, despite these benefits in monogastric animals, the effects of SH on rumen function remain unclear. Therefore, this study used an in vitro fermentation system to evaluate the effects of SH on ruminal ammonia nitrogen (NH_3_-N) concentration, bacterial crude protein (BCP) content, gas production, volatile fatty acid (VFA) production, and bacterial community composition.

## 2. Materials and Methods

### 2.1. Substrates and Treatments

SH was purchased from the market in Taiyuan City, Shanxi Province, China. SH is dark black in color. The effective component content is 28% fulvic acid and 29% free humic acid. The substrate was a high-concentrate diet; the ingredients and chemical composition of the basal diet are presented in Table 1.

In a completely randomized design, treatments involved supplementing 1 g (air-dry matter basis) substrates with different levels of SH (0 g/kg, 0.5 g/kg, 1 g/kg, and 2 g/kg). Moreover, a blank/control group was established, which contained neither the substrate nor SH. This blank group was utilized to account for the variations in rumen fluid used for in vitro fermentation and to determine the net gas production. Each experimental run consisted of four parallel experiments, and the entire experiment was replicated three times.

### 2.2. In Vitro Batch Culture

All procedures were approved by the Institutional Animal Care and Use Committee of Inner Mongolia Agricultural University (Protocol No. 2020069).

Four healthy female small-tailed Han sheep with similar body conditions, weighing 35.20 ± 1.45 kg and aged 6 months, were selected as rumen fluid donors. The experimental donor ewes were housed individually and fed a basic diet (with the same composition as shown in Table 1) at 08:00 and 18:00 daily, with free access to feed and water. Rumen fluid was collected using an oral stomach tube sampler connected to a vacuum pump (VP30, Beijing LabTech, Beijing, China) two hours prior to morning feeding. The initial saliva was discarded, and the receiving bottle was rinsed with a small amount of the original rumen fluid. The initial pH value of the rumen fluid was 7.13. The collected rumen fluid was filtered through four layers of gauze into an insulated bottle that was preheated to 39 °C and contained carbon dioxide. After collection, the insulated bottle was quickly transported to the laboratory and placed in a 39 °C water bath for insulation, with continuous introduction of carbon dioxide. According to Menke and Steingass [15], the rumen inoculum was mixed with a buffer solution in a ratio of 1:2 (*v*/*v*) with continuous flushing with CO₂. The mixed artificial rumen culture medium (~60 mL) was added to a culture bottle (100 mL) containing 1 g of substrate and incubated in a 39 °C incubator. After the bottle was sealed tightly, it was placed on a water bath shaker and incubated for 12 and 24 h.

### 2.3. Sample Collection and Chemical Analyses

After 12 and 24 h of fermentation, the bottles were placed in an ice bath at −10 °C to stop fermentation, and final pH was measured with a pH meter (FiveEasy Plus, Shanghai Mettler Toledo Co., Shanghai, China). The culture solution was placed in a 10 mL centrifuge tube and centrifuged at 4500 r min^−1^ for 15 min. The supernatant was separated and stored at −20 °C to determine the concentrations of BCP and NH_3_-N; the remainder was stored at −80 °C for analysis of VFAs.

### 2.4. Ruminal 16S rDNA Amplification, Sequencing, and Analysis

The DNA of the rumen microbiome in rumen fluid was extracted using an Omega Biotek kit (Norcross, GA, USA) and subsequently analyzed via 1.0% agarose gel electrophoresis. The V3-V4 regions of the 16S rRNA gene were amplified by PCR using universal primers 338F and 806R to characterize the rumen bacterial community. The PCR procedure was as follows: Each reaction mixture contained 15 µL of Phusion High Fidelity PCR Master Mix (New England Biolabs, Ipswich, MA, USA), 0.2 µM primers, and 10 ng of genomic DNA template. The amplification protocol consisted of an initial denaturation at 98 °C for 1 min, followed by 30 cycles of denaturation at 98 °C for 10 s, annealing at 50 °C for 30 s, and extension at 72 °C for 30 s. A final extension was performed at 72 °C for 5 min. The PCR products were detected using 2% agarose gel electrophoresis. Qualified PCR products were purified using magnetic beads, quantified by enzyme labeling, and pooled in equimolar amounts based on their concentrations. The pooled PCR products were then re-evaluated by 2% agarose gel electrophoresis. The target bands were excised, and the DNA was recovered using a universal DNA purification and recovery kit (TianGen, Beijing, China). The purified products were then subjected to high-throughput sequencing on the Illumina MiSeq platform (Version QIIME2-202202).

### 2.5. Statistical Analysis

Data were analyzed by one-way ANOVA using SPSS 26.0 software. Duncan’s multiple range test was used for multiple comparisons. Orthogonal polynomial analysis was conducted to assess the linear and quadratic effects of increasing SH levels on the respective indicators. A probability value of *p* < 0.05 (two-tailed) was considered statistically significant. Furthermore, intergroup differences in microbial composition were statistically analyzed using nonparametric *t*-tests. Significant differences were expressed as *p* < 0.05. When the synergistic effect was more pronounced, it resulted in more favorable outcomes for rumen fermentation and nutrient utilization. The multiple combination index (MFAEI) for different combinations is the sum of the single combination effect index (*SFAEI*) for different combinations (treatments) and can be used to determine the fermentation status of the rumen [16]. The calculation is as follows:

$$SFAEI=\frac{\sum_{m=1}^{n}(A-B)/n}{A}$$

*m*: Time of each fermentation point.

*n*: Total number of fermentation time points.

*A*: The values of each individual indicator in the experimental group at different cultivation time points.

*B*: The values of each individual indicator in the control group at different cultivation time points.

## 3. Results

### 3.1. Effect of SH Supplementation on NH_3_-N Concentration, BCP Content, and Gas Production

As shown in Table 2, after 12 h of incubation, the addition of different concentrations of SH had no significant effect on rumen pH, NH_3_-N concentration, and BCP content (*p* > 0.05). However, compared with the control group, 0.5 g/kg SH decreased GP by 11.66%, while 1 g/kg increased GP by 5.3% (*p* < 0.01).

After 24 h of incubation, the quadratic effect of SH supplementation decreased NH_3_-N concentration (*p* < 0.01) and increased BCP content (*p* < 0.05), while the linear effect increased gas production (*p* < 0.01). Compared with the control group, the SH1 group significantly decreased NH_3_-N concentration, while the SH2 group significantly increased NH_3_-N concentration (*p* < 0.01). The SH1 group had higher BCP content than the other groups (*p* < 0.05). Compared with the control group, the SH1 and SH2 groups significantly increased GP, while the SH0.5 group significantly decreased GP (*p* < 0.01). No significant differences in pH were observed among the groups.

### 3.2. Effect of SH Supplementation on Volatile Fatty Acids

The results from Table 3 illustrate that after 24 h of fermentation, SH supplementation linearly decreased (*p* < 0.05) the proportion of acetate compared with the control group.

### 3.3. Effect of SH Supplementation on SFAEI and MEAEI

As shown in Table 4, the MFAEI value of SH1 is the absolute maximum in this experiment, indicating that the appropriate concentration of SH for regulating rumen fermentation is SH1.

### 3.4. Effect of SH Supplementation on Rumen Bacterial Diversity

Taken together, we found that the addition of SH to the ration had a positive effect on rumen fermentation parameters, and the effect was optimal when SH was added at 1 g/kg. Therefore, we selected the CON and SH1 groups for bacteria analysis to reveal the effect of SH on rumen health and its mechanism.

**Figure 1:**
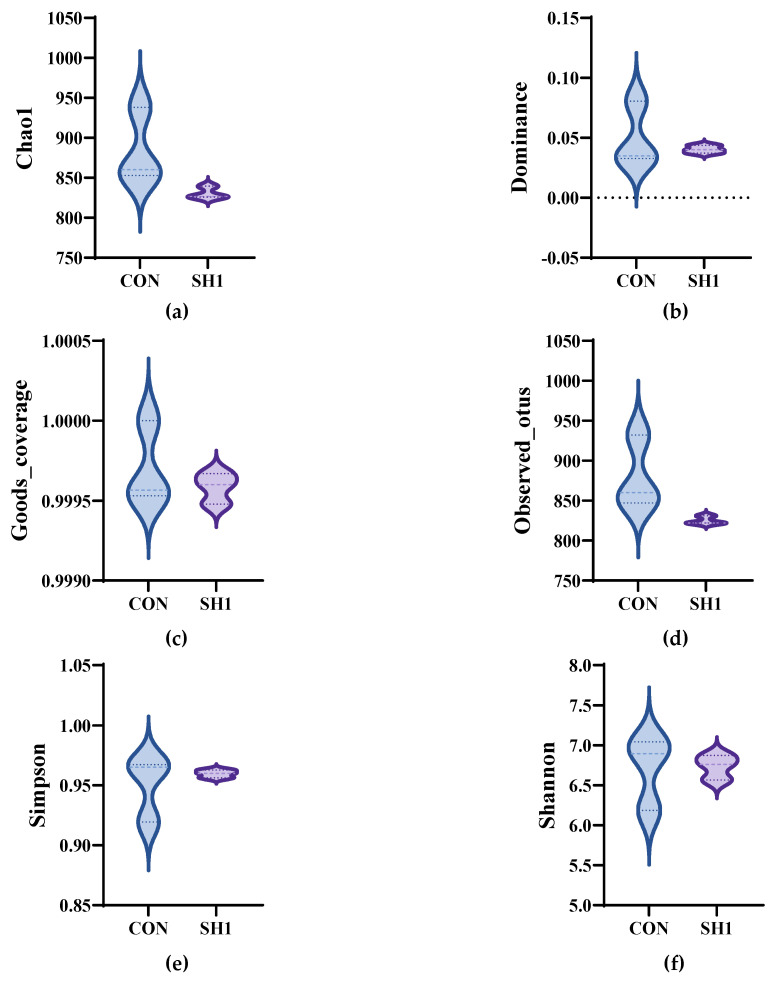
Effects of SH supplementation on the alpha diversity of the bacterial community in the rumen fluid of sheep. (**a**) Chao index; (**b**) Dominance index; (**c**) Goods_coverage; (**d**) Observed_otus; (**e**) Simpson index; (**f**) Shannon index. CON = without supplementation, SH1 = supplementation with 1 g/kg SH based on dry matter weight.

**Figure 2:**
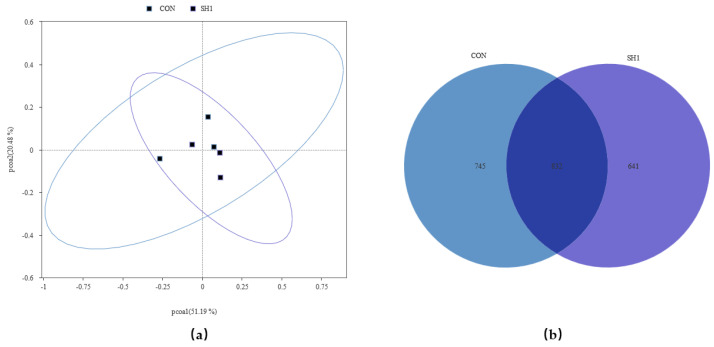
Effects of SH supplementation on the beta diversity of the bacterial community in the rumen fluid of sheep. (**a**) Bray–Curtis distance matrix PCoA of the bacterial community. (**b**) Venn Graph. CON = without supplementation, SH1 = supplementation with 1 g/kg SH based on dry matter weight.

A total of 543,013 original tags were produced, averaging 90,502 tags per sample. After size filtering, quality control, and removal of chimeras, 394,996 valid tags were retained, producing an average of 65,832 valid tags per sample, with a ratio of valid to raw data of 71.40% to 74.32%. The alpha diversity indices of the bacterial community, as depicted in Figure 1, demonstrated no significant variations (*p* > 0.05) across the Chao1, Dominance, Goods_coverage, Observed_otus, Shannon, and Simpson metrics. This suggests that the sequencing depth was adequate for the current investigation and that dietary supplementation with SH1 had no discernible impact on the richness or diversity of the rumen bacteria in the experimental subjects. The bacterial community composition was compared between the two treatment groups and analyzed using beta diversity analysis. As shown in the principal coordinate analysis (Figure 2a), the treatment and control groups were not clustered separately, indicating similar structures of the rumen bacteria. A Venn diagram (Figure 2b) depicted the overlap and unique ASVs between the two groups, with a total of 2218 ASVs identified.

### 3.5. Alteration in Rumen Microbial Composition Caused by SH

**Figure 3:**
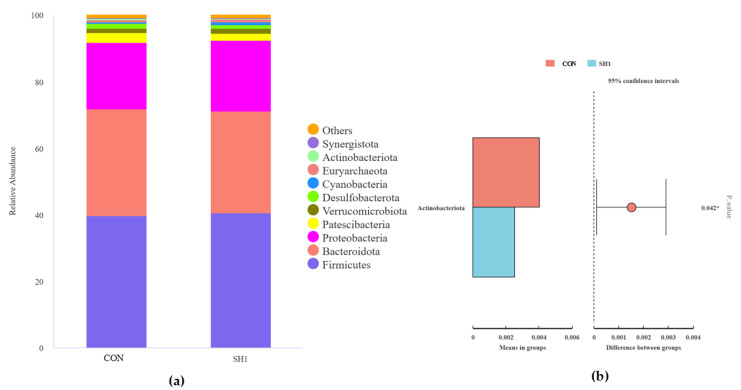

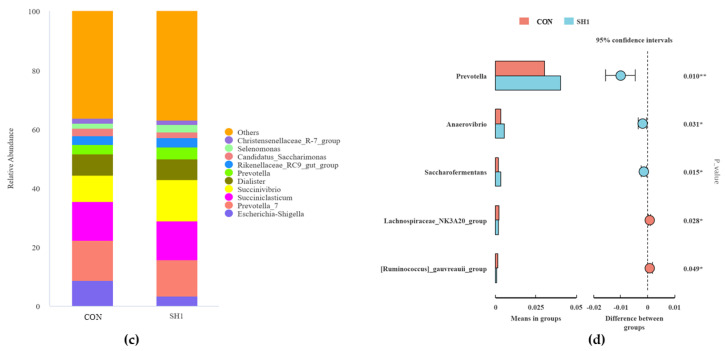
Effects of SH supplementation on ruminal bacteria at phylum and genus levels. The relative abundances (%) of bacterial phyla (**a**) and genera (**b**) in the in vitro rumen fermentation of CON and SH1. The *t*-test analysis of differential phyla (**c**) and genera (**d**). CON = without supplementation, SH1 = supplementation with 1 g/kg SH based on dry matter weight. (**a**) Relative abundance of phyla in fermentation broth after 24 h of fermentation and *t*-test analysis of differential bacteria (**b**). Relative abundance of genera in fermentation broth after 24 h of fermentation (**c**) and *t*-test analysis of differential bacteria (**d**). * *p* < 0.05, ** *p* < 0.01.

A total of 23 phyla and 205 genera were isolated and taxonomically classified in the current study. At the phylum level, Firmicutes, Bacteroidetes, and Proteobacteria were the dominant phyla, with a relative abundance greater than 1%; moreover, Firmicutes and Bacteroidetes have the highest relative abundance among all taxa, accounting for over 70% of the total abundance (Figure 3a). As shown in Figure 3b, the relative abundance of the phylum Actinobacteriota was higher in the CON group than in the SH1 treatment (*p* < 0.05). At the genus level (Figure 3c), 15 genera had a relative abundance greater than 0.1%, and *Prevotella_7, Succiniclasticum, Succinivibrio*, *Escherichia-Shigella*, and *Dialister* were the dominant genera. The two groups of genus-level *t*-test samples showed (Figure 3d) that in the SH1 group, the relative abundances of *Prevotella*, *Anaerovibrio*, and *Saccharofermentans* were significantly elevated (*p* < 0.05), while the relative abundances of *Lachnospiraceae_NK3A20_group* and *[Ruminococcus]_gauvreauii_group* were significantly reduced (*p* < 0.05) compared to the control group.

### 3.6. Correlation Analysis

**Figure 4:**
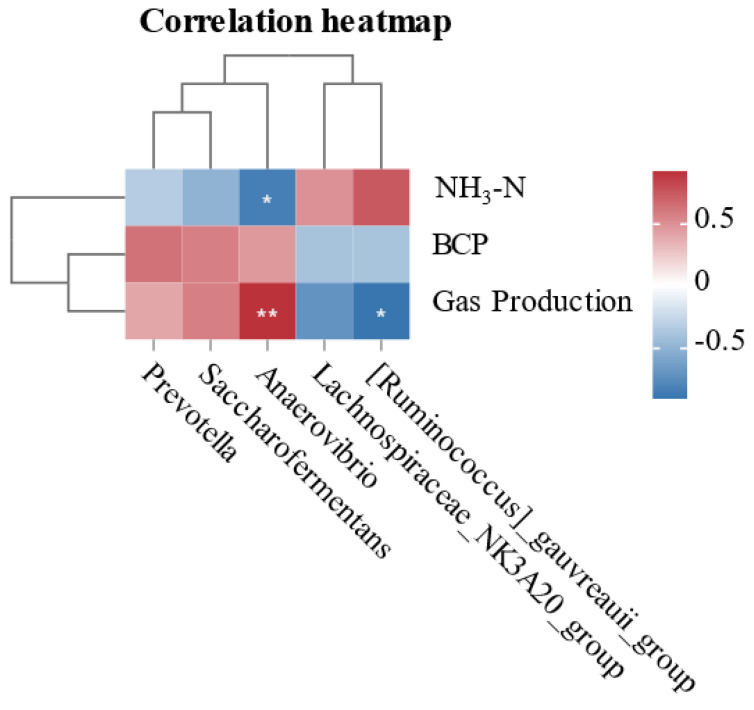
The heatmap of correlations between altered ruminal bacteria (genus level) and in vitro fermentation parameters. Positive and negative correlations are shown in red and blue, respectively. * *p* < 0.05, ** *p* < 0.01.

The correlation between rumen fermentation parameters and bacterial abundance differences during in vitro fermentation is shown in Figure 4. *Anaerovibrio* is positively correlated with NH_3_-N and negatively correlated with gas production. *[Ruminococcus] _gauvreauii_group* is negatively correlated with gas production.

## 4. Discussion

### 4.1. Rumen Fermentation

The pH value in ruminal pH varies from 5.5 to 7.5, which reflects the fermentation level of feed and the activity of microorganisms in rumen [17]. A low ruminal pH can impair ruminal fermentation function and affect the proliferation of fibrolytic bacteria [18]. The pH values of fermentation fluids showed no significant differences across various incubation periods and SH supplementation levels, demonstrating that SH administration did not markedly disrupt the ruminal pH homeostasis. This stability likely reflects the remarkable pH-buffering capacity inherent to rumen microbial ecosystems. It should be noted that the observed minor pH fluctuations might still exert biologically relevant effects on specific microbial populations, though these effects were not statistically significant under our experimental conditions. The lack of change in rumen pH in vitro or in vivo is also supported by previously reported results [19,20]. However, El-Zaiat et al. [13] found that the direct introduction of humate compounds into the rumen increased rumen pH in dairy goats. Differences between the current study and others can be attributed to species differences and the use of different experimental methods.

In the present study, supplementation with 1 g/kg SH significantly reduced the NH_3_-N concentration while increasing the BCP content in 24 h fermentation cultures compared to the control group. Notably, quadratic responses were observed for both parameters, with the NH_3_-N concentration showing a dose-dependent decrease and the BCP content demonstrating a corresponding increase at 24 h of fermentation. At the same time, the NH₃-N concentration decreased with the increase in bacterial populations, which suggests that NH₃-N was assimilated to support microbial growth. This reduction is driven by the nitrogen-binding activity of SH, which prolongs ruminal retention of NH₃-N [21], while concurrent bacterial uptake converts the retained NH₃-N into BCP through intracellular assimilation [22,23,24]. These dynamics align with fundamental rumen nitrogen metabolism, which begins when microbes degrade food protein or non-protein nitrogen into NH_3_-N, which is then transformed into BCP [25]. Microbial protein generated in the rumen provides an essential supply of amino acids for ruminants [26,27]. At the same time, BCP levels in the rumen usually reflect the growth rate and number of rumen microorganisms [28]. This phenomenon may be attributed to SH creating a more favorable microbial environment for utilizing nitrogen sources that enhances microbial NH_3_-N assimilation and conversion efficiency, consequently modulating both NH_3_-N dynamics and BCP synthesis parameters.

Gas production in the rumen is primarily the outcome of the fermentation of carbohydrates into VFA, and significant variations in carbohydrate fractions are reflected in the overall gas generated [29,30,31]. The cumulative gas production increased gradually with the increase in fermentation time and the gas production with the addition of 1 g/kg SH was the highest among all the groups. In this study, adding different doses of SH at different fermentation times had no effect on rumen total VFA, as well as acetate, propionate, and butyrate, and A:P. The results of other researchers are consistent with this fact [32,33]. This observation suggests that while SH supplementation modulates certain microbial activities during fermentation, it does not significantly alter the primary metabolic pathways or stoichiometric ratios of carbohydrate-derived VFA production. The microbial degradation and utilization of carbohydrates constitute a complex metabolic network, and although SH may induce subtle structural and functional adaptations in the microbial community, these modifications appear insufficient to disrupt the overall VFA production profile.

### 4.2. Rumen Bacteria

Following comprehensive evaluation of fermentation parameters, the experimental group supplemented with 1 g/kg SH and the basal diet group (control) were selected for 16S rRNA sequencing to investigate bacterial community dynamics. The rumen bacteria’s composition and activity have been demonstrated to have a dramatic impact on the host’s performance, health, and immune system [34,35]. The results revealed no significant difference in alpha diversity; this indicates that the sequencing depth was appropriate for the analysis and that SH supplementation had no effect on the bacterial community’s richness and diversity. Beta diversity resulted in identical clusters in the treatment and control groups, indicating similar compositions of bacteria in the rumen.

Ruminants have a very diverse bacterial community [36]. Firmicutes and Bacteroidetes are the major phyla in rumen, as previous research has indicated [37], and the results of our investigation corroborate this finding. Actinobacteriota usually have the ability to degrade complex organic matter and produce antimicrobial substances that help maintain the microbial balance in the rumen [38]. The genus *Prevotella* is thought to be the most prevalent group of ruminal bacteria [36,39], and representatives of this genus have been found to have a number of enzymes involved in the digestion of fiber [40,41]. Research on the genus *Anaerovibrio* in the rumen has focused primarily on its role in fat metabolism [42,43]. In particular, *Anaerovibrio lipolyticus* is considered one of the main lipolytic bacteria in the rumen and is capable of removing fat from the rumen for improved absorption and utilization, thereby enhancing the body’s efficiency in fat utilization [44]. Saccharofermentans can mainly promote cellulose decomposition [45], *[Ruminococcus]_gauvreauii_group* improves the digestive utilization of feeds [46]. Supplementation with 1 g/kg SH significantly reduced the relative abundance of Actinobacteriota at the phylum level, decreased *Lachnospiraceae_NK3A20_group* and *[Ruminococcus]_gauvreauii_group* at the genus level, and increased the relative abundance of *Prevotella_7, Succiniclasticum*, and *Succinivibrio*, compared with the control group. Correlation analysis showed that *Anaerovibrio* was negatively correlated with ammonia concentration and positively correlated with gas production. These findings suggest that *Anaerovibrio* may reduce NH_3_-N concentration while enhancing gas production during rumen fermentation through its metabolic activities. This phenomenon likely results from SH-induced modifications in microbial diversity and functional capacity, demonstrating that 1 g/kg SH supplementation can effectively modulate rumen bacterial community structure and consequently improve fermentation efficiency. In fact, microorganisms with a higher abundance proportion play an important role in the functioning of the rumen microbial ecosystem. However, a small portion of bacteria in the rumen community may have important ecological functions, but these functions have not been fully understood. One limitation of this study is the limited number of experimental replicates, which should be addressed in future experiments. In addition, bacterial diversity evaluated only at the 24 h time point may not be sufficient to reveal the potential relationship between bacteria and rumen fermentation parameters.

## 5. Conclusions

In summary, these findings demonstrate that dietary supplementation with SH at 1 g/kg improves rumen fermentation, enhances BCP synthesis, and modulates ruminal microbial composition. Therefore, the optimal inclusion rate for future animal studies appears to be 1 g of SH per kg of dietary dry matter in ruminant diets. However, further in vivo experiments should be conducted to determine the optimal SH dosage across different dietary types and growth stages in ruminants, evaluating its adaptive effects on rumen microbiota and animal production performance.

## Figures and Tables

**Table 1:** Ingredient and chemical composition of the basal diet (%, air-dry basis).

Items	Content
Ingredients (%)	
Corn grain	51.00
Soybean meal	12.00
Wheat bran	3.50
Sunflower shell	17.00
Cottonseed meal	3.50
Corn husk	2.00
DDGS	6.00
CaHCO_3_	0.30
Limestone	2.50
NaHCO_3_	0.30
NaCl	1.00
Premix ^1^	0.90
Total	100.00
Nutrient level ^2^	
Crude protein	14.11
Calcium, %	1.58
Phosphorous, %	0.86
Ash, %	7.51
Ether extract, %	2.63
Dry matter, %	90.11
Neutral detergent fiber, %	26.14
Acid detergent fiber, %	12.37

^1^ The premix provided the following per kg: vitamin A, 770,000 IU; vitamin D_3_, 160,000 IU; vitamin E, 780 IU; Fe, 1000 mg; Cu, 430 mg; Zn, 4100 mg; Mn, 160 mg; I, 17 mg; Se, 9 mg. ^2^ The Nutrient level is measured values.

**Table 2:** Effects of different fermentation times and SH additions on the pH value, NH_3_-N concentration, BCP content, and gas production of fermentation liquor.

Items	CON	SH0.5	SH1	SH2	SEM	*p*-Value		
						ANOVA	Linear	Quadratic
12 h								
pH	5.72	5.77	5.70	5.76	0.01	0.324	0.546	0.698
NH_3_-N (mg/100 mL)	12.71	14.29	14.25	12.98	0.47	0.390	0.932	0.093
BCP (mg/100 mL)	31.30	33.37	31.52	30.74	0.81	0.342	0.373	0.333
GP (mL)	66.45 ^b^	58.70 ^c^	69.98 ^a^	67.53 ^ab^	0.80	<0.01	0.052	0.412
24 h								
pH	5.70	5.66	5.66	5.68	0.02	0.263	0.679	0.062
NH_3_-N (mg/100 mL)	18.35 ^b^	19.08 ^ab^	15.36 ^c^	20.81 ^a^	0.51	<0.01	0.178	0.027
BCP (mg/100 mL)	33.02 ^b^	34.19 ^b^	38.25 ^a^	33.66 ^b^	0.71	0.032	0.629	0.011
GP (mL)	98.99 ^b^	89.13 ^c^	106.08 ^a^	104.37 ^a^	1.21	<0.01	0.002	0.455

NH_3_-N = ammonia nitrogen; BCP = bacterial crude protein; GP = gas production. SEM = standard error of the mean. CON, SH0.5, SH1, and SH2 are substrates supplemented with 0 g/kg, 0.5 g/kg, 1 g/kg, and 2 g/kg SH based on dry matter weight, respectively. ANOVA = contrast between CON, SH0.5, SH1, and SH2. ^a, b, c^ Within a row, values with different letter superscripts differ significantly at the *p* < 0.05 level. Linear = linear effect of SH addition; Quadratic = quadratic effect of SH addition.

**Table 3:** Composition and production of VFAs under different levels of SH supplementation.

Items	CON	SH0.5	SH1	SH2	SEM	*p*-Value		
						ANOVA	Linear	Quadratic
12 h								
Total VFAs (mmol/L)	3.61	3.72	3.56	3.65	0.04	0.518	0.991	0.819
Acetate (%)	35.89	36.19	37.53	36.08	0.18	0.517	0.923	0.138
Propionate (%)	34.07	34.14	34.31	34.09	0.12	0.087	0.228	0.783
Butyrate (%)	17.14	16.98	16.32	16.88	0.09	0.231	0.638	0.092
A/P	1.06	1.08	1.05	1.06	0.01	0.604	0.475	0.884
24 h								
Total VFAs (mmol/L)	3.95	3.98	3.98	4.00	0.02	0.839	0.413	0.796
Acetate (%)	33.82	33.55	33.68	32.95	0.17	0.136	0.028	0.481
Propionate (%)	31.18	31.58	31.77	31.39	0.16	0.600	0.736	0.183
Butyrate (%)	17.68	17.91	17.7	18.04	0.12	0.626	0.324	0.791
A/P	1.08	1.06	1.06	1.05	0.01	0.340	0.087	0.481

VFAs = volatile fatty acids; A/P = acetate/propionate. SEM = standard error of the mean. CON, SH0.5, SH1, and SH2 are substrates supplemented with 0, 0.5, 1, and 2 g/kg SH based on dry matter weight, respectively. ANOVA = contrast between CON, SH0.5, SH1 and SH2. Linear = linear effect of SH addition; Quadratic = quadratic effect of SH addition.

**Table 4:** SFAEI and MFAEI values under different SH concentrations.

Items	CON	SH0.5	SH1	SH2
SFAEI				
PH	0	−0.00094	−0.00558	0.001135
NH_3_-N	0	0.074266	−0.04362	0.029925
BCP	0	0.048186	0.071887	0.000404
GP	0	−0.09207	0.142658	0.102575
MFAEI	0	0.029443	0.165337	0.134039

SFAEI = single combination effect index; NH_3_-N = ammonia nitrogen; BCP = bacterial crude protein; GP = gas production; MFAEI = multiple combination index. CON, SH0.5, SH1, and SH2 are substrates supplemented with 0 g/kg, 0.5 g/kg, 1 g/kg, and 2 g/kg SH based on dry matter weight, respectively.

## Data Availability

The original contributions presented in this study are included in the article. Further inquiries can be directed to the corresponding author.
